# Supplementary material for: Investigation of the Effect of Exendin-4 on Oleic Acid-Induced Steatosis in HepG2 Cells Using Fourier Transform Infrared Spectroscopy
Source: Biomedicines. 2022 Oct 20;10(10):2652. doi: 10.3390/biomedicines10102652 (PMC9599706; doi:10.3390/biomedicines10102652)
Supplement: Supplementary file 1 [file biomedicines-10-02652-s001.zip › biomedicines-1756644-supplementary.pdf]

Supplementary data

Investigation of the effect of Exendin-4 on oleic acid-induced steatosis in HepG2 cells using Fourier Transform Infrared Spectroscopy

Olfa Khalifa, Ehsan Ullah, Kamal H. Mroue, Raghvendra Mall, Nayla S. AL-AKI, Abdelilah Arredouani

Supplementary Table S1: The spectral assignments and protein components with their assigned wavelength in IR region.

**Infrared band**	**Integrated spectral range (cm^{-1})**
**Lipid Components**	
**CH_2 symmetric stretching**	2852–2800
**CH_2 asymmetric stretching**	2915–2930
**CH_3 asymmetric stretching**	2950–2960
***C-H stretching**	2994–2800
**Olefin =CH**	3000–3027
**Carbonyl ester (C=O) stretching**	1745–1731
**Protein Components**	
**Amide I**	1700–1600
**Amide II**	1555–1535
**Amide III**	1350–1200
**Amide I Secondary structure (1700–1600)**	
**α helix**	1660–1650
**Random coil**	1645–1630
**Parallel β-sheet**	1610–1635
**Beta- shoulder (1665–1695)**	
**β -turn**	1680
**Anti-parallel β-sheet**	1695

*** Total lipid region**
